# Case Report: Creeping Growth in Lymphoplasmacyte-Rich Meningioma—A Radiologic Variant

**DOI:** 10.3389/fsurg.2021.775560

**Published:** 2021-12-14

**Authors:** Jiuhong Li, Xin Zan, Min Feng, Xueyun Deng, Si Zhang, Wenke Liu

**Affiliations:** ^1^Department of Neurosurgery, West China Hospital, Sichuan University, Chengdu, China; ^2^West China School of Medicine, Sichuan University, Chengdu, China; ^3^Department of Pathology, West China Hospital, Sichuan University, Chengdu, China; ^4^Department of Neurosurgery, The Second Clinical Medical College of North Sichuan Medical College, Nanchong Central Hospital, Nanchong, China

**Keywords:** creeping-growth, lymphoplasmacyte-rich meningioma, mechanism, lymphatic system, case report

## Abstract

Lymphoplasmacyte-rich meningioma (LRM) is a rare histologic subtype of meningioma. Creeping-growth pattern is uncommon in meningioma, and the mechanism is unclear. Here, we report a 44-year-old man presented with extremities weakness for 2 months and incontinence for 2 weeks. Head and neck MRI revealed diffuse creeping-growth nodular meningeal masses with skull base, tentorium, sella area, and C1-6 vertebral plane involvement. An operation was carried out, cervical and lower clivus part of the lesion was resected, but gross total resection could not be achieved due to the widespread lesions. Pathologic examination revealed the diagnosis of LRM. The patient is free from progression clinically 3 months postoperatively. We also conducted a systematic literature review about LRM with creeping-growth pattern. A total of only nine cases (including the present case) of creeping-growth LRMs were included and analyzed in terms of clinical manifestations, radiological features, treatment, and outcome. LRMs show a higher rate (7.5%) of creeping-growth pattern than other types of meningiomas. The average creeping length of all creeping-growth LRMs was 11.4 ± 10.9 cm (range, 3–30 cm). Most cases (66.7%) had obvious peritumoral edema. Total removal rate is low (33.3%), and two of them (22.2%) received biopsy, followed by steroids treatment (or further immunosuppressive drugs therapy) and radiotherapy. The recurrence rate is higher than conventional LRMs (22.2 vs. 11.3%), and one patient (11.1%) died 11 months after treatment. Creeping-growth pattern in LRM may be considered as a general radiologic variant. The recurrence rate is higher compared with LRM with round/swelling pattern. We speculated that the pathogenesis of creeping growth in LRM may be associated with damage of lymphatic systems of the central nervous system.

## Introduction

Intracranial meningioma represents the most common primary brain tumor (1). Radiologically, they frequently behave as swelling dura-based masses (2). Meningiomas with creeping-growth pattern are a rare occurrence and are frequently misdiagnosed preoperatively (3–5). Lymphoplasmacyte-rich meningioma (LRM) is probably the rarest histologic subtype of meningioma, accounting for around 1% of all intracranial meningiomas (6, 7). It is characterized by

infiltration of lymphoplasmacyte that overshadows the meningioma components (8). We reported a patient with LRM who presented with diffuse creeping-growth nodular meningeal masses with skull base, tentorium, sella area, and C1-6 vertebral plane involvement radiologically. Eight additional cases of creeping-growth LRM were identified in the literature and were also presented. The mechanism of creeping growth in LRM is unclear. Therefore, we also made a hypothesis to elucidate the mechanism of this rare growth pattern in LRM based on lymphatic systems of the central nervous system (CNS).

## Case Presentation

**Figure 1:**
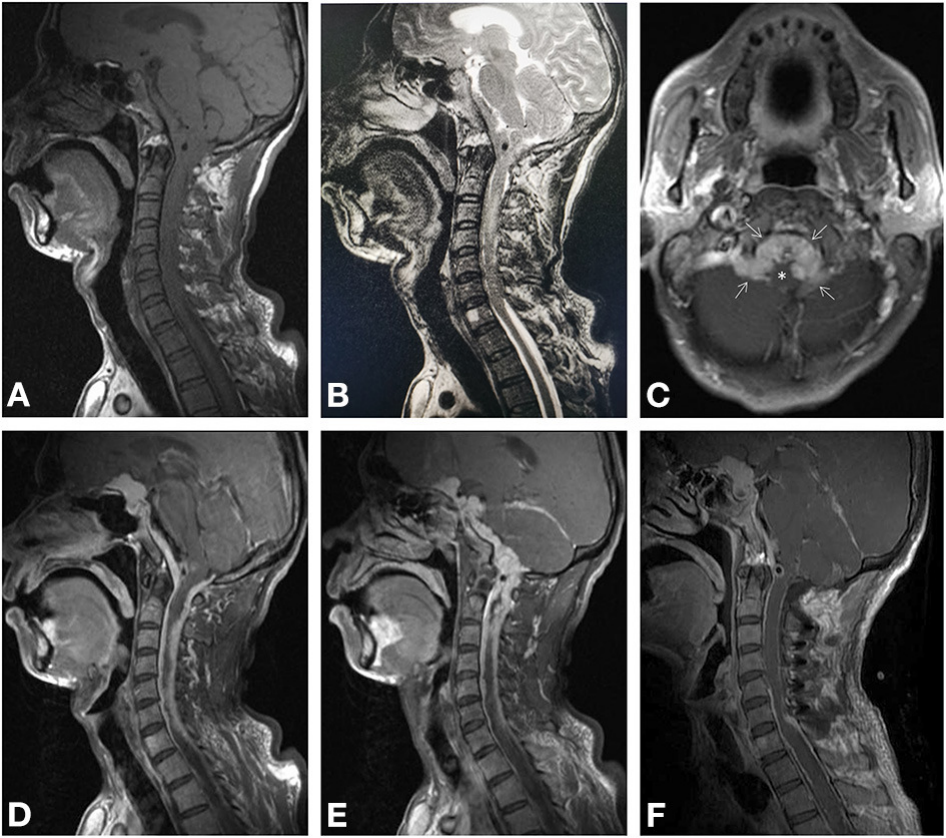
Magnetic resonance imaging. Preoperative imaging revealed isointense lesions on T1-weighted **(A)** and T2-weighted imaging **(B)**, compressing the spinal cord. Axial enhanced T1-weighted imaging **(C)** revealed enhanced lesion located at midline premedullary cistern and lateral medullary cisterns (*arrows*), compressing the brainstem (*asterisk*). Sagittal enhanced T1-weighted imaging **(D, E)** showed multiple nodular lesions continuously creeping through the tentorium and meninges of sellar region, skull base, and C1–C6. Postoperative-enhanced MRI **(F)** proved the spinal and lower region of clivus of the tumor was totally removed.

**Figure 2:**
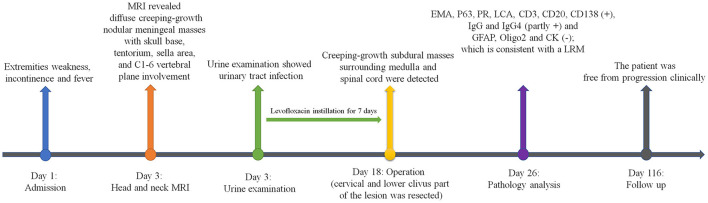
Timeline of the case presentation.

**Figure 3:**
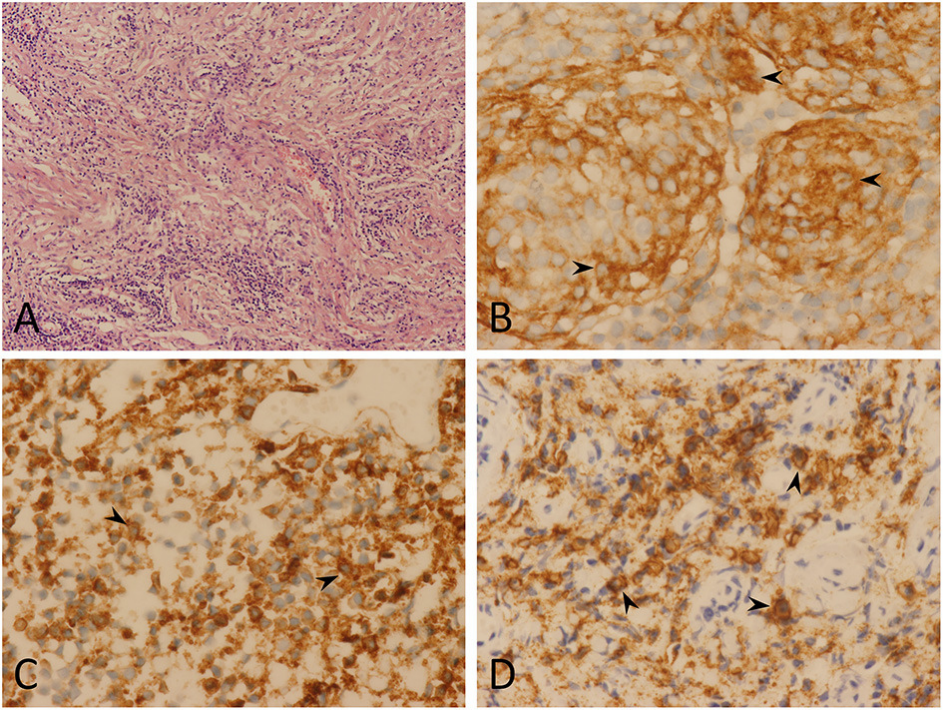
Pathological findings. Hematoxylin and eosin (magnification, ×100) **(A)** showing lymphoplasmacytes infiltrated the tumor stroma, overshadowing the meningioma component. Immunohistochemistry testing showed epithelial membrane antigen positive (*arrowheads*) (magnification, ×400) **(B)**, CD20 positive (*arrowheads*) (magnification, ×400) **(C)**, and CD138 positive (*arrowheads*) (magnification, ×400) **(D)**. In conclusion, pathological findings indicate a lymphoplasmacyte-rich meningioma.

A 44-year-old man presented with extremities weakness for 2 months and incontinence for 2 weeks. He had a history of hospitalization in another hospital 1 month ago. The electromyography testing results of limbs were normal and brain plain MRI showed no obvious abnormalities. A diagnosis of symptomatic parkinsonism (encephalitis-induced most likely) was made, and Madopar was prescribed for 2 weeks, but the symptoms did not alleviate and incontinence occurred. Thus, an indwelling urinary catheter was placed. The dosage of Madopar was gradually increased, but the symptoms did not alleviate. Two weeks later, he was transferred to our hospital. His temperature was 37.6°C on admission. Physical examination disclosed decreased muscle strength of extremities (muscle strength grade III for the left and IV for the right) and Babinski sign positive for lower limbs. White blood count was normal; HIV testing was negative. Head and neck MRI showed creeping-growth isointense lesions on T1- and T2-weighted images, compressing the spinal cord (Figures 1A,B). After administration of gadolinium, diffuse creeping-growth nodular meningeal masses with skull base, tentorium, sella area, and C1-6 vertebral plane involvement were detected, compressing medulla and adjacent spinal cord (Figures 1C–E). Routine urine examination of the patient showed that the white blood cell count was 44/HP, and pus cells were detected. The patient received levofloxacin instillation for 7 days and the temperature went normal. A preliminary diagnosis of meningeal inflammatory lesion was made based on radiological manifestations. Subsequently, he underwent an operation. The patient was placed with a left lateral decubitus position, and we used a posterior midline approach. We formed the bone flap (within 4 cm above foramen magnum) and removed posterior arch of C1, performed laminotomy of C2–C6. During operation, creeping-growth subdural masses surrounding the medulla and the spinal cord were detected, with a clear boundary. We mainly used ultrasonic aspiration to achieve tumor debulking and micro-scissors to achieve separation. The cervical and lower clivus part of the lesion was resected; however, gross total resection could not be achieved due to the widespread lesions. Finally, long-segment laminoplasty of C2–C6 and cranioplasty of occipital bone were performed. Postoperative MRI confirmed the total resection of the cervical and lower clivus part of the lesion (Figure 1F). The muscle strength of extremities of the patient recovered to normal level, and the Babinski sign was negative for lower limbs 4 days after surgery. Then he discharged and was transferred to our two-way referral hospital. The patient is free from progression clinically 3 months postoperatively (Figure 2). A histological test revealed that the tumor stroma was surrounded by prominent lymphocytes and plasmocytes. The tumor cells were positive for EMA, P63, PR, and negative for GFAP, Oligo2, and CK; lymphocytes were positive for LCA, CD3, and CD20; plasmocytes were positive for CD138, partly positive for IgG and IgG4, which is consistent with an LRM (Figure 3). A multidisciplinary team (Neurosurgery, Neurology, Oncology, and Pathology team) discussion for the patient was performed, and we recommended adjuvant radiotherapy while the patient refused. Thus, we recommended regular imaging follow-up, and if the tumor had sign of progression, stereotactic radiotherapy should be performed and the patient agreed.

## Literature Review

This study was conducted and reported according to the Preferred Reporting Items for Systematic Reviews and Meta-Analysis statement.

### Search Strategy and Article Selection

The PubMed and Embase databases were searched for case reports and series relevant to LRM published up to December 31, 2020. “Lymphoplasmacyte,” “rich,” “lymphoplasmacyte-rich,” and “meningioma” were used as either keywords or medical subject headings. In addition, the references cited in selected articles were hand-searched and reviewed to identify additional potentially eligible studies. Titles and abstracts of all articles were screened. Only articles describing cases of creeping-growth LRM were selected. Articles without any radiological imaging were excluded; articles published in languages other than English were also excluded from the study. References from relevant reviews were hand-searched. Subsequently, relevant articles were retrieved and evaluated independently by two authors (JL and XZ). A cross-reference check of the citations of each included relevant article was done to ensure that no relevant studies were missed by the computerized database search. Disagreements regarding the inclusion of studies were resolved by the judgment of a third author (SZ).

### Data Extraction

Two authors (JL and XZ) independently extracted the following characteristics from the included studies: setting, study type, demographic data of presented patients, duration, tumor location, radiologic findings, treatment, outcome, and follow-up. The length of creeping growth in cases with complex skull base involvement is determined by the maximal creeping length on the axial, coronal, and sagittal planes.

## Result

Our search identified 46 potential citations for full-text evaluation. Eight cases of creeping-growth LRM from five different publications between 1997 and 2018 could be extracted from the literature and matched the inclusion criteria of our study.

Till now, around 120 cases of LRM have been reported in the English literature, and nine cases (7.5%) of creeping-growth LRM (including the present case) have been reported (4, 5, 9–12). The key characteristics of all reported creeping-growth LRM are summarized in Supplementary Table 1. There were five male and four female patients. The mean age was 40.8 ± 12.8 years (range, 22–55 years). Unlike other histologic subtypes of meningiomas, no female predominance was observed (0.8:1), which was similar to the literature report (4, 11). Most cases (55.6%) were non-skull base masses (Supplementary Table 1). The average creeping length of all creeping-growth LRMs was 11.4 ± 10.9 cm (range, 3–30 cm). Most cases (62.5%) had obvious peritumoral edema, higher than that (40%) of round LRMs reported by Tao et al. (11). Total removal rate is low (33.3%) due to the wide-spread lesion. Two of them (22.2%) received biopsy, followed by steroids treatment (or further immunosuppressive drugs therapy) and radiotherapy. The recurrence rate is higher than conventional LRMs (22.2 vs. 11.3%) and one patient (11.1%) died 11 months after treatment (11).

## Discussion

Lymphoplasmacyte-rich meningioma was firstly described by Banerjee and Blackwood in 1971, which was characterized by infiltration of lymphoplasmacyte that overshadows the meningioma components (8). It is probably the rarest histologic subtype of meningioma, accounting for around 1% of all intracranial meningiomas and was classified as WHO grade I tumor (6, 7). The pathogenesis, radiologic features, and outcome of this subtype of meningioma remain unclear because of their rarity. Here, we report an extremely rare case of LRM with a creeping-growth pattern mimicking idiopathic hypertrophic pachymeningitis and examine previously reported cases of creeping-growth LRM in an attempt to provide an up-to-date summary of the condition and illustrate the possible mechanism of this rare kind of growth pattern.

## Differential Diagnosis

For subdural masses with creeping growth, the most common preoperative diagnosis can be a subdural hematoma, lymphoma, dural metastasis, and sarcoma. However, our study suggests that meningiomas should also be included in the differential diagnosis. Subdural LRMs usually have “dural tail sign” and are more likely to show extensive uneven thickening of nearby meninges and peritumoral edema (4). Patients with subdural hematoma usually have a brain-trauma history, with surrounding edema on T2-weighted image and no enhancement on enhanced MRI. Subdural lymphomas tend to be primary ones such as mucosal-associated lymphoid tissue lymphomas, usually with nodular/lobulate borders and leptomeningeal extension (13–17). Dural metastasis lesions usually demonstrate hyperintensity on T2-weighted imaging and have a history of systemic primary neoplasm, especially from melanoma, breast, and prostate cancers (16, 18, 19). Sarcomas tend to affect pediatric or younger patients and are usually accompanied by systemic leukemia (20). Other differential diagnoses include empyema or granuloma, neurosarcoidosis, tuberculosis, inflammatory pseudotumor, idiopathic hypertrophic pachymeningitis and Rosai–Dorfman disease (5, 12–14, 16, 18, 19, 21, 22). Thus, when dealing with subdural masses, neurosurgeons should at least be reminded of the possibility of LRM, and further examinations such as enhanced MRI should be considered which would be helpful for the differential diagnosis.

## Therapeutic Strategy

As for treatment of creeping-growth LRM, surgical resection is the optimal treatment modality to achieve pathological diagnosis, alleviate symptoms, and sometimes cure the disease (4, 5). However, the total removal rate is very challenging in some cases because of the wide-creeping lesion, which is hard to expose and resect (4, 5, 9, 10). As summarized in Supplementary Table 1, two patients had recurrence, and they both received incomplete resection. Thus, we hold that for patients receiving incomplete resection, radiotherapy should be recommended to achieve better tumor control. Hirunwiwatkul et al. (5) performed a biopsy to achieve pathological diagnosis of an LRM extending from planum sphenoidale down along the clivus to the foramen magnum and then prescribed prednisone and later azathioprine, followed by fractionated radiotherapy. And the patient was free from recurrence for over 6 months. Therefore, we hold that for creeping-growth LRM, a comprehensive treatment program (surgery, steroids, and immunosuppressive treatment combined with radiotherapy) could be recommended, especially for those receiving incomplete resection.

## The Lymphatic System of CNS

In 2015, Louveau et al. (23) discovered functional lymphatic vessels lining the venous sinuses in mice, which could drain both fluid and immune cells from cerebrospinal fluid–interstitial fluid flow. Absinta et al. (24) then demonstrated the existence of true lymphatic vessels in humans by non-invasive MRI, which run parallel to the venous sinus and alongside branches of the middle meningeal artery. Meningeal lymphatic vessels, together with the perineural sheaths surrounding cranial and spinal nerves and arachnoid granulations are thought to be three distinct kinds of outflow sites of cerebrospinal fluid in recent years (25). Meningeal lymphatic systems are mainly located within the dura mater and carry fluid, macromolecules, and immune cells, such as plasmocytes and lymphocytes, to the draining deep cervical lymph nodes (26).

## Possible Mechanism of Creeping-Growth Pattern in LRM

Radiologically, meningioma frequently manifests as swelling dura-based mass (1, 2). The creeping-growth pattern of meningiomas is rare; however, LRMs show a higher rate (7.5%) of creeping-growth pattern than other types of meningioma (1, 27). The mechanism of creeping growth in LRM is unclear. Yamaki et al. (12) hold that microscopically tumor component of LRM is almost replaced by abundant inflammatory cells; thus, LRM may behave differently from conventional meningioma radiologically. Liu et al. (28) suggest that lymphoplasmacytes could form cuff-like structure around the vascular tissue and damage the blood–brain barrier, thus causing enhancement on post-contrast MRI. We initially elucidate this rare phenomenon based on the lymphatic systems of the CNS.

We speculated that the mechanism of creeping growth in LRM might be as follows: (1) meningioma grows and erodes the meningeal lymphatic systems; (2) meningioma may even keep growing and transmitting to adjacent dura mater through the highway of the lymphatic system. (3) meningiomas express chemotactic agents such as inflammatory and chemotactic cytokines to assemble lymphocytes and plasmocytes; (4) vast lymphocytes and plasmocytes subsequently keep leaking from the lymphatic structure, overshadowing the meningioma components; and (5) lymphocytes and plasmocytes change the microenvironment of the tumor and prevent the gathering and adhering of meningioma components or destroy/hamper the formation of peritumoral fibrin, restraining the swelling growth of the tumor, and thus LRM tends to grow diffusely along the meninges. In our case, the meningioma grows as continuous multiple nodular pattern probably under the influence of prominent infiltrated lymphoplasmacytes instead of forming a regular shape (swelling/round) of a meningioma (Figures 1A–E). In addition, the margin of case 4 (Supplementary Table 1) reported by Luo et al. is quite near the superior sagittal sinus and transverse sinus (4). Furthermore, case 2 (Supplementary Table 1) showed the vicinity of the tumor and occipital sagittal sinus (4). These cases indicated that the pathogenesis of LRM might be associated with the lymphatic system, especially the wider lymphatic vessels alongside intracranial venous sinuses. For the case treated in our hospital reported by Yang et al. (9), the whole intracranial dura mater was involved, which indicated the meningioma components may be transmitted through the meningeal lymphatic system to reach such widespread meninges. As for those LRMs behaving as swelling pattern, the destruction of the meningeal lymphatic systems may occur while the tumor is relatively large, whereas the infiltrated immune cells could not reshape the tumor.

The hypothesis that the pathogenesis of LRM associates with CNS lymphatic system damage could interpret the following four aspects of LRM behaviors. (1) Massive lymphocytes and plasmocytes infiltrating the tumor is uncommon, which means they are most likely to originate from lymphatic systems rather than blood vessels or cerebrospinal fluid. (2) Many creeping-growth LRMs could reach a creeping length of over 10 cm. The extraordinary diffuse involvement of dura mater may be attributed to the lymphatic system pathway transfusion. (3) It could explain why LRM may behave as regular (swelling/round) shape or a relatively higher rate (around 10%) of creeping-growth pattern than other histologic subtypes of meningiomas due to the different stages of lymphatic system damage. (4) The lymph proteins leaking from lymphatic systems or the dysfunction of cerebrospinal fluid absorbing caused by lymphatic destruction could further induce brain edema, which is compatible with the high rate (62.5%) of severe peritumoral edema in creeping-growth LRM (29).

The strength of our study is that we reviewed all the cases of creeping-growth LRMs and concluded that LRMs show a higher rate of creeping growth compared with other histological meningiomas; thus, the creeping-growth pattern in LRMs may be considered as a common radiologic variant. In addition, we proposed a hypothesis that the pathogenesis of LRMs may be associated with damage of lymphatic systems, which could nicely explain why LRM may behave as regular (swelling/round) shape or a relatively higher rate (around 10%) of creeping-growth pattern than other histologic subtypes of meningiomas due to the different stage of lymphatic system damage. The limitations of our study are retrospective in nature. Furthermore, because of financial limitations and local medical insurance policy restrictions, we could not obtain sufficient data regarding genetic conditions. Moreover, some cases of the literature were lost to follow-up. Finally, no evidence from the experiments confirming the mechanism of creeping growth of LRM existed. Therefore, further investigations, such as tracing the origin of lymphoplasmacytes, analyzing the function of infiltrated lymphocytes and plasmocytes, the possible chemotactic agents expressed by LRM, and exploring genetic differences, are necessary to elucidate the preferred mechanisms of creeping growth in LRM.

## Conclusion

Lymphoplasmacyte-rich meningiomas show a higher rate (7.5%) of creeping-growth pattern than other types of meningiomas and may be considered as a general radiologic variant. Currently, though total removal is challenging, surgical resection is the optimal treatment to cure the disease and relieve the symptoms. The recurrence rate is higher compared with LRM with round/swelling pattern. We proposed a hypothesis that the pathogenesis of creeping growth in LRM may be associated with damage of the lymphatic systems of the CNS.

## Data Availability Statement

The original contributions presented in the study are included in the article/Supplementary Material, further inquiries can be directed to the corresponding authors.

## Ethics Statement

The studies involving human participants were reviewed and approved by Ethics Committee of West China Hospital of Sichuan University. Written informed consent was obtained from the patient and his legal representatives for the publication. Written informed consent was obtained from the individual(s) for the publication of any potentially identifiable images or data included in this article.

## Author Contributions

XZ, SZ, and WL contributed to the conception and design of the study. JL, MF, and XD developed the methodology. JL, XZ, MF, XD, SZ, and WL helped in the acquisition and analysis of data. JL, XZ, and MF involved in writing, reviewing, and revision of the manuscript. MF and WL provided the technical and material support. SZ and WL supervised the study. All authors have read and approved the manuscript. All authors contributed to the article and approved the submitted version.

## Funding

This work was supported by the Youth Program of the National Natural Science Foundation of China (Nos. 81701174 and 81801178); the Program of Science & Technology Department of Sichuan Province (No. 2020YFS0222).

## Conflict of Interest

The authors declare that the research was conducted in the absence of any commercial or financial relationships that could be construed as a potential conflict of interest.

## Publisher's Note

All claims expressed in this article are solely those of the authors and do not necessarily represent those of their affiliated organizations, or those of the publisher, the editors and the reviewers. Any product that may be evaluated in this article, or claim that may be made by its manufacturer, is not guaranteed or endorsed by the publisher.

## Abbreviations

CNS: central nervous system
LRM: lymphoplasmacyte-rich meningioma.
