# Supplementary material for: Case Report: Creeping Growth in Lymphoplasmacyte-Rich Meningioma—A Radiologic Variant
Source: Front Surg. 2021 Dec 14;8:775560. doi: 10.3389/fsurg.2021.775560 (PMC8712332; doi:10.3389/fsurg.2021.775560)
Supplement: Supplementary file 1 [file Table_1.docx]

**Table 1.** **Key Characteristics of Reported Cases of Creeping-growth Lymphoplasmacyte-rich Meningioma**

| **Authors & year** | **Pt. No.** | **Age**  **(years)/sex** | **Duration**  **(months)** | **Clinical features** | **Location** | **Length, cm** | **Peritumoral**  **edema** | **Treatment** | **Outcome** | **Follow-up**  **(months)** |
| --- | --- | --- | --- | --- | --- | --- | --- | --- | --- | --- |
| Yamaki et al. 1997 [12] | 1 | 22/Male | 120 | Visual disturbances,  hearing impairment, sensory disturbance | Foramen magnum to C-5 | ~12 | NA | PR | Recurrence, 7 years | 84 |
| Luo et al. 2015 [4] | 2 | 54/Female | NA | NA | Left frontal temporalis | 4 | Yes | TR | No recurrence | 31 |
| Luo et al. 2015 [4] | 3 | 30/Male | NA | NA | Right lateral ventricle | 4 | Yes | TR | No recurrence | 34 |
| Luo et al. 2015 [4] | 4 | 52/Female | NA | NA | Occipital sagittal sinus | 6 | Yes | STR | Recurrence, 1 year | 56 |
| Luo et al. 2015 [4] | 5 | 39/Female | NA | NA | Right frontal cupular part | 3 | Yes | TR | No recurrence | 42 |
| Cha et al. 2016 [10] | 6 | 55/Male | 1 | Left-sided weakness and ataxia | Left tentorium | 4 | Yes | NTR | NA | NA |
| Hirunwiwatkul et al. 2007 [5] | 7 | 24/Male | Several years | Headache, visual loss | Planum sphenoidale  to foramen magnum | 10 | No | Biopsy, steroids, immunosuppressive drugs and radiotherapy | No recurrence | 6 |
| Yang et al. 2018 [9] | 8 | 47/Female | 6 | Occipital-cervical region pain，hearing loss, upper limbs numbness | Entire intracranial dura mater | ＞30 | No | Biopsy, radiotherapy, steroids | Dead | 11 |
| Present  case | 9 | 44/Male | 2 | Extremities weakness, incontinence | Skull base, tentorium, sella area, and C1-6 | ＞30 | No | PR | No Recurrence | 3 |

*NA* not available, *NTR* near total removal, *PR* partial removal, *Pt. No.* patient number, *STR* subtotal removal, *TR* total removal.
